# A Bibliometric Meta-Analysis of Colistin Resistance in *Klebsiella pneumoniae*

**DOI:** 10.3390/diseases9020044

**Published:** 2021-06-20

**Authors:** Ozioma Forstinus Nwabor, Pawarisa Terbtothakun, Supayang P. Voravuthikunchai, Sarunyou Chusri

**Affiliations:** 1 Division of Infectious Diseases, Department of Internal Medicine, Faculty of Medicine, Prince of Songkla University, Hat Yai, Songkhla 90112, Thailand; nwaborozed@gmail.com (O.F.N.); pawarisa.3tk@gmail.com (P.T.); 2 Division of Biological Science, Faculty of Science, Prince of Songkla University, Hat Yai, Songkhla 90112, Thailand; supayang.v@psu.ac.th

**Keywords:** colistin resistance, *Klebsiella pneumoniae*, chromosomal mediated resistance, plasmid-borne resistance, bibliometric analysis

## Abstract

Colistin is a last resort antibiotic medication for the treatment of infections caused by carbapenem-resistant *Klebsiella pneumoniae*. In recent years, various mechanisms have been reported to mediate colistin resistance in *K. pneumoniae*. This study reports a bibliometric analysis of published articles retrieved from the Scopus database relating to colistin resistance in *K. pneumoniae*. The research trends in colistin resistance and mechanisms of resistance were considered. A total of 1819 research articles published between 1995 and 2019 were retrieved, and the results indicated that 50.19% of the documents were published within 2017–2019. The USA had the highest participation with 340 (14.31%) articles and 14087 (17.61%) citations. Classification based on the WHO global epidemiological regions showed that the European Region contributed 42% of the articles while the American Region contributed 21%. The result further indicated that 45 countries had published at least 10 documents with strong international collaborations amounting to 272 links and a total linkage strength of 735. A total of 2282 keywords were retrieved; however, 57 keywords had ≥15 occurrences with 764 links and a total linkage strength of 2388. Furthermore, *mcr*-1, colistin resistance, NDM, *mgrB*, ceftazidime-avibactam, MDR, combination therapy, and carbapenem-resistant *Enterobacteriaceae* were the trending keywords. Concerning funders, the USA National Institute of Health funded 9.1% of the total research articles, topping the list. The analysis indicated poor research output, collaboration, and funding from Africa and South-East Asia and demands for improvement in international research collaboration.

## 1. Introduction

Bacterial resistance to chemotherapeutic antibiotics has become a global public health threat. The current annual death rate associated with infections caused by drug-resistant microorganisms is estimated at 700,000 and might increase to 10 million by 2050 if urgent action is not taken [1]. In 2017, the WHO global priority list classified drug-resistant pathogens into three tiers, with carbapenem-resistant *Enterobacteriaceae* as critical and requiring immediate attention [2]. The increased prevalence of resistance to carbapenems associated with the expression of specific virulence factors and the spread of new clones, limits treatment options for infections caused by multidrug-resistant *Enterobacteriaceae* such as *Klebsiella pneumoniae* [3,4]. This has led to the reintroduction of polymyxins which were previously discontinued for use in humans [5]. Colistin, also known as polymyxin E, is an antibacterial cationic polypeptide that binds to the negatively charged lipid A of Gram-negative outer membrane lipopolysaccharide. The antibacterial agent competitively displaces membrane-stabilizing divalent cations (Ca^2+^ and Mg^2+^) from the lipopolysaccharide, resulting in cell membrane disruption and cell death [6]. Gram-negative bacteria are known to be intrinsically susceptible to colistin except for bacteria of the genera Proteus, Providencia, Morganella, Serratia, Edwardsiella, and Burkholderia [6]. Colistin is a last-resort antibiotic therapy against multidrug-resistant infections caused by Gram-negative carbapenem-resistant bacteria; however, the overuse of colistin has resulted in the acquisition of resistance by several bacterial genera. 

The emergence of colistin-resistant *Enterobacteriaceae* poses a severe challenge to the continued reliance on antibiotics chemotherapy. The spread of carbapenemase-producing colistin-resistant *K. pneumoniae* has been reported by researchers from different regions of the world, including Africa [7,8,9], Asia [10,11,12], Australia [13], Europe [14,15], North America [16], and South America [17]. Recently, a 71.3% prevalence was reported for colistin resistance among Escherichia coli, and *K. pneumoniae* isolates from Thailand [18]. Up to 2015, colistin resistance was related to chromosomal mutations resulting in the alteration of Gram-negative outer membrane lipopolysaccharide, which reduces the negative charge of the lipid A component and affinity for colistin. However, in 2016 the emergence of a plasmid-borne *mcr*-1 gene, a gene of the phosphoethanolamine transferase enzyme family was first reported [19,20,21,22]. This has raised concerns due to the potential rapid dissemination of resistant mediating plasmid between strains and subsequent selection pressure. 

Colistin resistance in *K. pneumoniae* is mediated by several factors, including alterations of capsular polysaccharide and capsular type, efflux pumps, outer-membrane alterations, and alterations in lipid A and lipopolysaccharides. The inactivation of the mgrB gene, by products of the pmr operon encoding a negative feedback regulator of PhoQ-PhoP signaling system, is majorly responsible for acquired chromosomal related colistin resistance. Upregulation of PhoQ-PhoP activates the Pmr system responsible for lipopolysaccharide modification [23,24]. Alterations in the *mgrB* gene coupled with the disruption of pmrH operon, mutation in *phoQ*, elevated expression of *phoPQ*, and mutations of the crrAB two-component regulatory system with elevated expression of *pmrCAB* were reported in colistin-resistant *K. pneumoniae*. In addition, genes involved in cation transport and maintenance of membrane integrity were upregulated [25]. Studies have shown that the interruption of transcripts and amino acid mutation in mgrB are major mechanisms contributing to colistin resistance [26]. Amino acid substitutions in *K. pneumoniae* CrrB protein were associated with resistance to colistin [27]. A hospital outbreak of colistin-resistant carbapenemase-producing *K. pneumoniae* was traceable to the clonal expansion of an mgrB deletion mutant of a ST512 strain [28]. Furthermore, the presence of insertion sequences and nonsense/missense mutations on the cell chromosomal components were responsible for the inactivation of the mgrB gene [29]. Insertion sequences in the mgrB gene or surrounding region of *crrCAB* disrupted the regulatory function of the gene [30]. An IS-like element at the nucleotide position 75 of the mgrB and substitutions in PhoQ were identified as mechanisms of resistance [31]. In addition, an epidemiological investigation of colistin-resistant *K. pneumoniae* strains revealed that capsular type K64 and ST11 are the prevalent capsular and sequence types amongst colistin-resistant strains [26]. Atomic force microscopy revealed an altered capsule in a susceptible strain and intact capsules in a resistant strain suggesting that capsular polysaccharides influenced the response of *K. pneumoniae* to colistin [32]. Similarly, the presence of an efflux pump attributed to mutations in the two-component systems induced a high resistance to colistin in sequence type ST147 *K. pneumoniae* [33]. 

The spread of plasmid-mediated colistin resistance presents a critical threat. The carriage of resistant mediating genes on self-transmissible broad-host-range plasmid accentuates the potential to spread to a wider range of pathogens. Although the *mcr* plasmid that mediates colistin resistance was first reported between 2015–2016, recent research findings suggest a wide range of spread amongst *Enterobacteriaceae*, including *K. pneumoniae* and *E. coli* with the emergence of several variants. The *mcr*-2 to *mcr*-9, share 81%, 32.5%, 34%, 36%, 83%, 35%, 31% and 36% identical amino acid sequences with *mcr*-1, respectively [34,35,36]. In addition, minor variants have been reported for *mcr*-2, -4, -5, -6, -7, -8 and -9; however, a greater number of minor variants were identified for *mcr*-1 and *mcr*-3 with 18 and 28 variants, respectively [37]. 

A study aimed at investigating the prevalence of the *mcr*-1 in clinical *E. coli* and *K. pneumoniae* isolates in Thailand reported that 1.4% of *K. pneumoniae* isolates harboured the *mcr*-1 gene. The minimum inhibitory concentrations of colistin for the resistant isolates ranged between 4 and 64 mg/L [18]. Also, among 64 isolates of colistin-resistant *E. coli* and *K. pneumoniae* obtained in Iran, 1.7% harboured the *mcr*-1 gene [38]. Recently, a novel variant of the *mcr*-1 named *mcr*-1.2 associated with the pIncX4 plasmid was detected [39]. Also, an *mcr*-1.2 gene encoding a Gln3–Leu functional variant of *mcr*-1 was detected in ST512 *K. pneumoniae* isolates obtained from a rectal swab of a leukemic child. The variant *mcr*-1.2 gene was carried on a transferable IncX4 plasmid [40]. The *mcr*-gene has been reported in environmental and food samples, which calls for prompt surveillance and intervention to limit its spread. The *mcr*-1 gene was reported in bacterial isolates from meat samples collected from European countries, including Poland, Germany, and Czech Republic. Isolation of microorganisms from the meat samples yielded forty-two isolates carrying the *mcr*-1 gene with *E. coli* (*n* = 39) and *K. pneumoniae* (*n* = 3) [41]. Screening of retail fruits in China revealed the presence of *mcr*-1 carrying *E. coli* and *K. pneumoniae* on fruits surfaces [42], whereas in India *K. pneumoniae* isolated from raw food samples showed the presence of the *mcr*-1 gene [43]. The presence of resistant genes along the food chain presents a substantial risk for the dissemination of plasmid-mediated colistin resistance in meat and fresh-cut products. Furthermore, the colistin resistance gene *mcr*-1 was found in a *K. pneumoniae* strain of sequence type 313 recovered from hospital sewage [44]. Norman and co-workers reported the presence of clinically relevant antibiotic-resistant genes, including the plasmid-mediated *mcr*-1 gene in wastewater treatment plants in Germany [45]. Metagenomic analysis of wastewater sampled from 9 locations in Germany revealed the presence of the *mcr*-1 gene. The authors further reported a high prevalence of other gene variants with a relative abundance of *mcr*-1, *mcr*-3, *mcr*-4, *mcr*-5, and *mcr*-7 [46]. In addition, *K. pneumoniae* isolated from a stool sample from healthy volunteer in Northern Thailand harboured both *mcr*-1 and *mcr*-3 with a MIC of 16 mg/L [47]. Novel variants of *mcr*-3 named *mcr*-3.21, *mcr*-3.26, and *mcr*-3.28, located on plasmids IncP1, IncFII, and IncI1 type have been identified in *K. pneumoniae* strains from Laos and Thailand. The genetic environment of the *mcr*-3.21 and *mcr*-3.26 genes was composed of a composite transposon [37]. Whole Genome Sequencing of three *K. pneumoniae* isolates from chickens in China identified a novel variant of colistin resistance gene *mcr*-7.1 that shared a 70% amino acid identity with the *mcr*-3 gene. The *mcr*-7.1 gene was found in an IncI2-type plasmid (pSC20141012) that co-harboured the blaCTX-M-55 gene in one isolate [48]. The emergence of various variants of the *mcr*-gene is suggested to be driven by unknown selective pressure within the environment, animals, and humans [49]. In China, *mcr*-8, was found located on a transferrable 95,983-bp IncFII-type plasmid in *K. pneumoniae*. The *mcr*-8 showed 31.08%, 30.26%, 39.96%, 37.85%, 33.51%, 30.43%, and 37.46% identity to *mcr*-1, *mcr*-2, *mcr*-3, *mcr*-4, *mcr*-5, *mcr*-6, and *mcr*-7, respectively [50]. An *mcr*-8.1 variant discovered from a whole genome sequencing in Lebanon, was carried in a conjugative ~300 kb multi-replicon plasmid having IncFIA, IncR and IncHI1B [51]. *K. pneumoniae* isolates harbouring the *mcr*-8 gene and other resistant genes, including blaOXA-48, blaCTX-M-15 β-lactamases was reported by a whole-genome sequence study in Algeria [52]. Similarly, plasmid-mediated gene *mcr*-8.1 variant was recovered from infected patients in Bangladesh. The transferable *mcr*-8.1 was harboured in an identical highly stable multidrug-resistant IncFIB(pQil) plasmid of ~113 kb, which belonged to an epidemiologically successful *K. pneumoniae* clone, ST15 [53]. In addition, whole-genome sequencing of *K. pneumoniae* strains revealed 4 colistin-resistant strains that harboured the *mcr*-8.2 variant that differed from *mcr*-8.1 by four amino acid substitutions. The *mcr*-8.2 was located on a nonself-transmissible plasmid containing IncQ, IncR, and IncFII replicon [54]. Likewise, a plasmids co-harbouring both tmexCD1-toprJ1 pump responsible for tigecycline resistance and *mcr*-8 plasmid and *bla_NDM_*-harbouring IncX3 plasmid was isolated from humans in a nationwide surveillance [55]. Novel colistin-resistant gene *mcr*-9 has been reported from *Klebsiella oxytoca*, an opportunistic human pathogen that causes nosocomial infection and *Klebsiella quasipneumoniae* subsp. *Quasipneumoniae* from Qatar and Latin America, respectively [56,57]. The co-occurrence of multiple transferable plasmids within an isolate is a threat to public health and might further stretch the burden of antimicrobial resistance by developing pandrug-resistant isolates. The novel variant *mcr*-10 was found in Enterobacter roggenkampii with a 79.69% nucleotide identified with *mcr*-9. The *mcr*-10 gene encodes a protein with 82.93% identical amino acids with *mcr*-9 and conferred 4-fold increase in colistin MIC. The authors reported that the *mcr*-10 gene was located adjacent to a site-specific recombinase-encoding gene and was bracketed by IS903, and thus may be mobilized by site-specific recombination or composite transposon [58].

As antimicrobial resistance spreads, it has become essential to monitor the trends, patterns, and prevalence of resistance emergence and spread across the globe. This will provide reliable information and will enable proper surveillance. Thus, the present study summarizes the current scientific research literatures. It collates data on the reported mechanisms and prevalence of colistin resistance in *K. pneumoniae*. The study aims to provide quantitative analysis and statistics on the trends of publications on the subject matter over time and explore the structure of networking amongst researchers and countries in the field. This will help to identify areas of weakness such as countries and regions with research gaps and deficiencies, and thus inform researchers and public health policy makers of the realities as reflected in research outputs. Using bibliometric analysis, research gaps, risk areas, and key players (authors, institutions, funding agencies, and countries) in the field can be identified.

## 2. Results 

**Figure 1:**
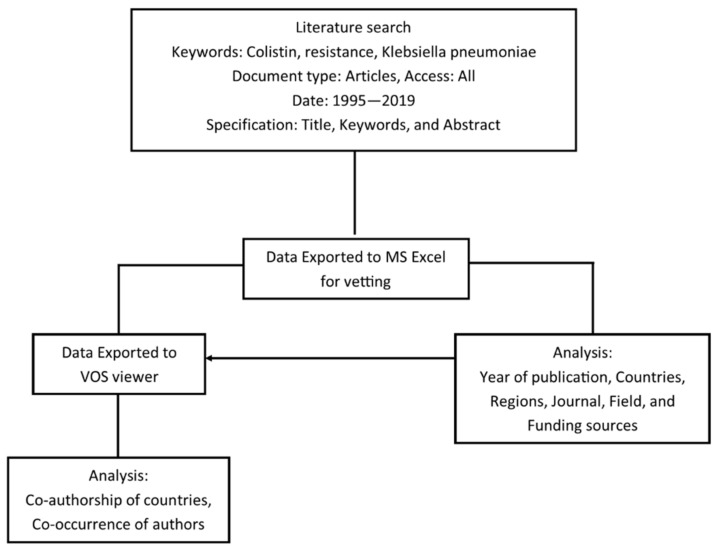
Process flowchart for meta-analysis of data retrieved from the Scopus database.

A meta-analysis of the data obtained from 1995–2019 on the Scopus database was performed by bibliometrics. The retrieved data was analyzed based on the year of publication, countries and regions, co-authorship of countries, co-occurrence of authors keywords, and funding sources. Analysis parameters were chosen to highlight the key drivers of research relating to colistin resistance in *K. pneumoniae*. Figure 1 presents the process flowchart employed in the study. Table 1 presents a collection of colistin-resistant mechanisms published in the literature from 1995 to June 2020, and Table 2 presents the reported prevalence of colistin resistance in *K. pneumoniae* from different countries.

### 2.1. Trend of Publication by Year

**Figure 2:**
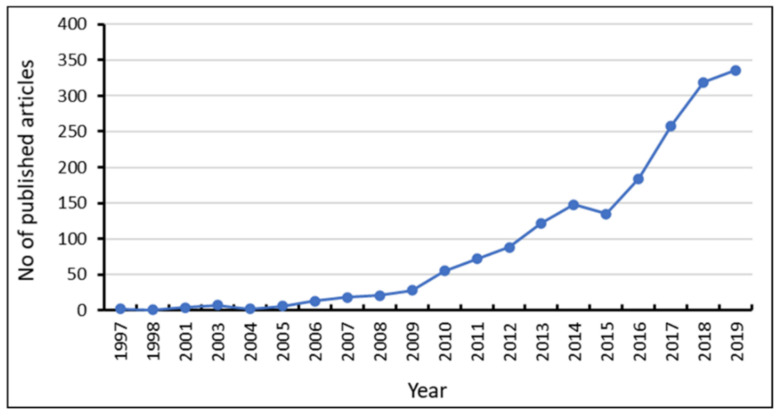
Yearly research trends on colistin-resistant *Klebsiella pneumoniae*.

A total of 1819 research articles relating to colistin resistance in *K. pneumoniae* were exported from the Scopus database into VOS viewer software. The results indicated that early articles relating to the topic were published from 1997, as no preceding document was found (Figure 2). The results suggested a progressive increase in literatures pertaining to colistin resistance, with a steep increase from 2016 to 2019.

### 2.2. Distribution of Publications Based on Region and Country

**Figure 3:**
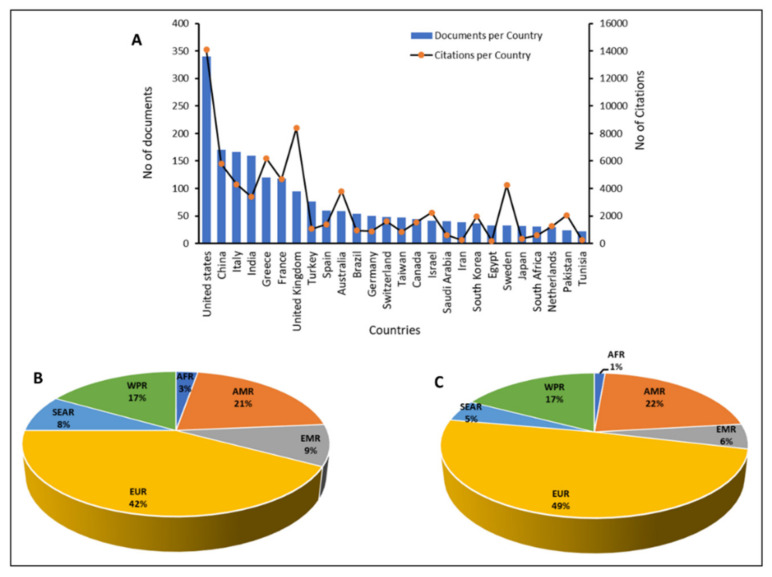
Country and regional participation in research related to colistin resistance in *K. pneumoniae.* AFR: African Region; AMR: American Region; EUR: European Region; WPR: West Pacific Region; EMR: Eastern Mediterranean Region; SEAR: Southeast Asia Region. (**A**): shows publications and citation based on countries; (**B**): shows publications based on WHO global epidemiological regions; (**C**): shows citations based on WHO global epidemiological regions.

Research outputs on colistin resistance in *K. pneumoniae* were analyzed based on the number of publications and citations per country. It was observed that 99 countries had published contributions on colistin resistance in *K. pneumoniae*. Figure 3A shows the publication and citation stratification for countries with at least 20 publications relating to the topic. The results revealed that the 26 countries with a minimum of 20 publications accounted for 82.83% of all the published documents and 91.22% of citations. The United States of America had the highest contribution with 340 published research articles and 14,087 citations, amounting to 14.31 and 17.61%, respectively. China contributed 170 published articles with 5790 citations, equal to 7.15 and 7.24%, respectively. The top European countries included Italy, Greece, France, and the United Kingdom, while Egypt, South Africa, and Tunisia represented the Africa continent. Based on citations, the United Kingdom contributed 10.5%, second to the United States of America. Stratification of documents based on WHO global epidemiological regions indicated the following: that the European Region contributed (1002; 42%); American Region (496; 21%),; West Pacific Region (397; 17%); Eastern Mediterranean Region (219; 9%); Southeast Asia Region (197; 8%); and the African Region (65; 3%) (Figure 3B). Based on citations, the European Region contributed (39,264; 49%); America Region (17,465; 22%); West Pacific Region (13,848; 17%); Eastern Mediterranean Region (4466; 6%); Southeast Asia Region (3881; 5%); and the African Region (1076; 1%) (Figure 3C). The results supported the conclusions of the world health organization that “information on the true extent of antimicrobial resistance in the African region is limited due to poor surveillance and scarcity of accurate and reliable data on antimicrobial resistance in general, and on antibacterial resistance in particular” [107]. Similarly, a recent study aimed at characterizing the global distribution and diversity of *mcr*-9 plasmid reservoirs noted that the plasmid was distributed in 21 countries across six continents. Stratification based on the continent revealed a larger number of *mcr*-9 positive isolates in Europe (*n* = 72; 52.2%), and lower numbers in South America (*n* = 1; 0.7%) and Africa (*n* = 1; 0.7%). The authors noted that the global prevalence of *mcr*-9 might be underestimated due to lack of epidemiological investigations [108]. 

### 2.3. Co-Authorship of Countries

**Figure 4:**
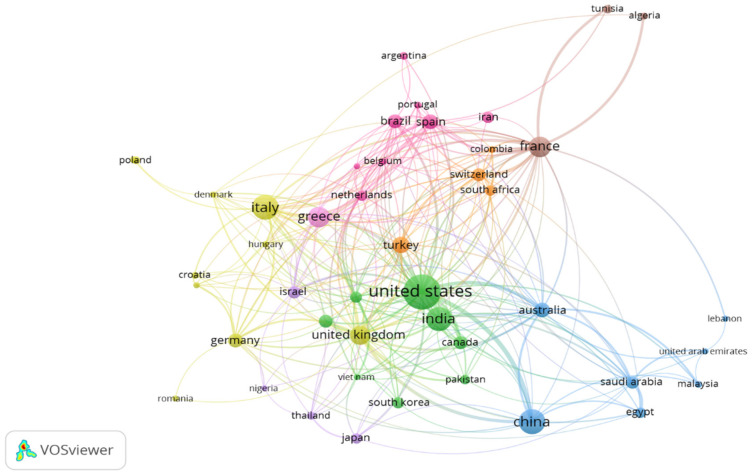
Co-authorship network of countries with at least 10 publications on colistin resistance in *K. pneumoniae*-related research within 1995 and 2019.

Co-authorship of countries analysis helps to understand the international research collaborations in the field of colistin resistance in *K. pneumoniae*. Data retrieved from the Scopus tdatabase was exported to VOS viewer software and analyzed at ≤10 countries per document for countries with at least ten published articles. The result indicated that 45 countries fitted the parameter settings distributed into 9 clusters, 272 links, and a total linkage strength of 735. The network visualization (Figure 4) showed that the United States of America had the largest collaboration network distributed into 2 clusters, 33 links, and a total linkage strength of 194 with 343 documents. The relative strength of the collaboration, measured by the thickness of the connecting lines between countries, [109] revealed that the USA had the highest collaboration with China and Australia. Research collaborations within Europe were high, with France in the lead, involved in 8 clusters, 31 links, total linkage strength of 114, and a total document of 121. Switzerland, Germany, the UK, Spain, Italy, Greece, and the Netherlands were the top European countries with elaborate research collaboration. From the Asian continent, China showed elaborate international collaborations, and was involved in 3 clusters, with 15 links, total linkage strength of 70, and 171 documents. India was involved in 2 clusters, 16 links, and atotal linkage strength of 42, and 161 documents. The African continent was led by South Africa with 7 clusters, ten links, total linkage strength of 19, and 31 documents. The results suggested poor international collaboration between Africa and Southeast Asia and the international community. The overlay visualization (Figure S1) presented the countries with the most currently published articles relating to colistin resistance in *K. pneumoniae*. The result indicates that Japan, Turkey, Switzerland, Vietnam, Portugal, Thailand, and Egypt are currently involved in research relating to the topic.

### 2.4. Co-Occurrence of Authors Keywords

**Figure 5:**
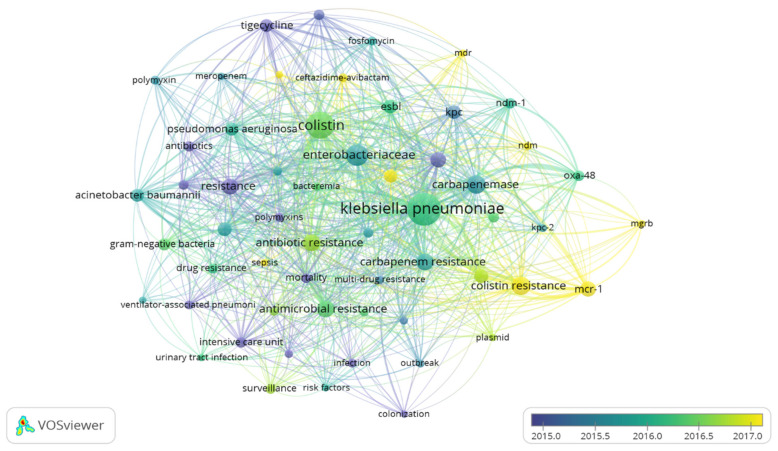
Network of authors keywords with at least 15 co-occurrences on colistin-resistance in *K. pneumoniae*-related research within 1995 and 2019.

Keywords used by the authors were retrieved and analysed based on occurrence and co-occurrence. A total of 2282 keywords were retrieved; however, to ensure that only the most frequently occurring keywords were used, the analysis was restricted to keywords with at least 15 occurrences. Analysis based on the specified parameters highlighted 57 keywords classified into 6 clusters based on relatedness, 764 links, and a total linkage strength of 2388. The most occurring keywords were, *K. pneumoniae*, colistin, *Enterobacteriaceae*, colistin-resistance, *mcr*-1, carbapenemase, carbapenem resistance, resistance, and antimicrobial resistance. *K. pneumoniae* had a total occurrence of 302, with 52 links and a total linkage strength of 555 whereas colistin and colistin resistance had an occurrence of 205 and 95, respectively. Colistin resistance was associated with 5 clusters, 34 links, and a total linkage strength of 148, whereas colistin showed involvement with 3 clusters 52 links and a total linkage strength of 397. *mcr*-1 and mgrB were both associated with all 5 clusters, *mcr*-1 had 27 links, a total linkage strength of 133 and 69 occurrences, while mgrB had 13 links, and a total linkage strength of 38 with 15 occurrences. Other keywords included “carbapenem-resistant *Enterobacteriaceae*”, plasmid, multidrug resistance, NDM, combination therapy, and antibiotic resistance. The co-occurrence of keywords showed the relatedness of individual keywords and frequency of co-usage. The overlay visualization of co-occurrence of keywords shows the trend and evolution of keywords to reflect the dynamism of keywords with respect to shifts in research interest andthe emergence of new issues such as mechanisms and antibiotics approval. The analysis indicated that keywords such as carbapenems, carbapenemase, carbapenemases, mortality, tigecycline, polymyxin, and antibiotics were popularly used between 2015 and 2016, and the years preceding. However, keywords including *mcr*-1, colistin resistance, NDM, mgrB, ceftazidime-avibactam, MDR, combination therapy, and carbapenem-resistant *Enterobacteriaceae* are currently trending as indicated (Figure 5). This signifies the current progress in antimicrobial resistance and recent emerging resistant mechanisms.

### 2.5. Funding Agencies

**Figure 6:**
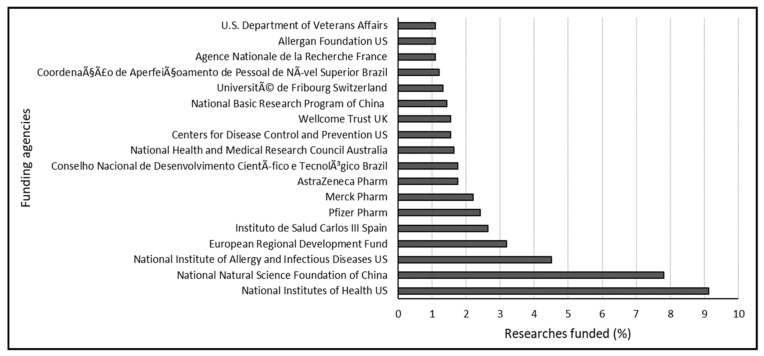
Research funders with at least 10 funded documents on colistin resistance in *K. pneumoniae*-related research between 1995 and 2019.

To understand the research trend, the data obtained from the Scopus database was analyzed based on funders. This helps picture the involvement of government institutions, agencies, and companies such as the pharmaceutical industries in driving the antimicrobial search and research. The results revealed the involvement of 159 funders with 909 funded research outputs. Analysis of the top funding agencies with a minimum of 10 funded documents indicated that 18 agencies funded 47.41 % of the research with 431 documents (Figure 6). The top 5 leading funders werethe National Institutes of Health US, National Natural Science Foundation of China, National Institute of Allergy and Infectious Diseases US, European Regional Development Fund, and Instituto de Salud Carlos III Spain with 9.1% (83 documents), 7.8% (71 documents), 4.5% (41 documents), 3.2% (29 documents), and 2.6% (24 documents), respectively. Some of the companies involved in research funding included Pfizer, Merck, AstraZeneca Pharma, Basilea Pharma, Meso Scale Diagnostics, Gilead Science, Allergan, Astellas Pharma, and Novartis. However, Pfizer, Merck, and AstraZeneca Pharma topped the list with contributions of 2.42% (22 documents), 2.20% (20 documents), and 1.76% (16 documents), respectively. Independent non-governmental charitable organizations involved in research funding included Wellcome Trust UK with a contribution of 1.54% (14 documents) and Allergan Foundation USA with a contribution of 1.10% (10 documents). It is also worth pointing out that the United States topped the list of funders with 3 national representatives amongst the top 18 funders including, National Institutes of Health, the National Institutes of Allergy and Infectious Diseases, and the Center for Disease Control and Prevention.

## 3. Discussion

The revitalization of colistin as the re-emerging choice drug for the management of infections caused by carbapenem-resistant Gram-negative bacteria faced a sudden setback following the emergence of resistance in *Enterobacteriaceae*. This has left the health sector vulnerable to assault from carbapenem-resistant bacterial isolates, leading to increased morbidity and mortality. *K. pneumoniae*, an opportunistic bacterial species associated with mild to severe infections is a potential threat due to the recent emergence and spread of hypervirulent strains that havebroadened the number of people susceptible to infections, including both healthy and immunosufficient individuals [110]. Currently, researchers are overwhelmed in the search for alternative effective options, with synergistic combination therapies serving as an interim option [111]. This bibliometric analysis of research publications relating to colistin resistance in *K. pneumoniae* presents an overview of the research trends. The date obtained indicated the availability of articles relating to colistin and *K. pneumoniae* between 1997 and 2006. However, the first published multiclonal cluster of colistin resistance in *K. pneumoniae* on the Scopus database was reported by a retrospective observational study conducted in a Greek hospital intensive care unit which reported minimum inhibitory concentrations >4 mg/L for *K. pneumoniae* isolates [112]. Preceding articles published between 2005 and 2006 presented colistin as the re-emerging antibiotic of choice for the management of drug-resistant Gram-negatives including *K. pneumoniae*. Although colistin is effective against Gram-negative carbapenemase-producing *Enterobacteriaceae*, the re-emergence and revitalization of colistin were met with the sudden development of resistance following the first detection of carbapenem-resistant *Enterobacteriaceae*. Analysis of research output relating to colistin-resistant *K. pneumoniae* indicates a progressive increase beginning in 2009. However, 50.19% of all the retrieved documents were published within the last three years (2017–2019). This is due to the surge in cases of colistin resistance resulting from the extensive use and misuse of colistin in chemotherapy, and veterinaries, leading to an increased severity of colistin resistance as well as the heightened interest prompted by the categorization of antimicrobial resistance and prioritization of carbapenem-resistant *Enterobacteriaceae* in the critical class of the WHO global priority pathogens list [2]. In addition, the steep rise over the last three years stems from the elevated interest due to the emergence and rapid spread of colistin resistance arising from the emergence of the transferrable plasmid-mediated *mcr*-genes first reported in 2016, and its variants. Moreover, the acquisition of resistance in environmental isolates and reports of resistant mediating genes in both food and water might have widened the horizon of researchers interested in the topic, thus contributing to the rapid growth of research output. In addition, our results further suggested poor research outputs from the Eastern Mediterranean, Southeast Asia, and Africa, which calls for improved investment and surveillance on antimicrobial resistance. The available data on the prevalence of antimicrobial resistance across the globe indicated high levels in Africa and Asia. The yearly death attributable to AMR by 2050 according to the review on antimicrobial resistance estimates 4,150,000 and 4,730,000 annual deaths for Africa and Asia respectively [113]. The low research output on antimicrobial resistance suggests that irrespective of the World Health Assembly global and national action plan on AMR, there might be a lack of awareness and intervention policies within countries in the regions. Global AMR maps viewed at resistancebank.org indicated a high rate of resistance in Asia and Africa, with colistin resistance hotspots in Asia, and Latin America. Furthermore, resistance maps reveal the absence of major colistin resistance hotspots in Africa, and this probably suggests low surveillance and research activities regarding resistance. A recent work on the global trends in antimicrobial resistance in animals in low- and middle-income countries identified Asia as possessing the largest hotspot of AMR [114]. Similar to the findings of Sweileh and Moh’d Mansour [115], the results of the present study indicated that the United States and China dominated the research on colistin resistance in *K. pneumoniae*. In this study, China and India which are hotspots of AMR ranked 2nd and 4th in research output. The high rate of AMR in China has been linked to the unregulated usage of antimicrobial drugs in food-producing animals [116]. This might likewise be applicable in neighbouring countries such as India and other Asian countries. In addition, the dissemination of resistant genes through the trade route in food products, especially imported animal products are possible factors which could promote the spread of resistance. Our results further showed that the United States and China besides producing the highest number of publications also led in funding research on the topic.

To the best of our knowledge, this work presents initial data on bibliometric analysis of colistin resistance in *K. pneumoniae*. However, although the authors tried to ensure that the information presented represents the true trend as obtained from the Scopus database and vetted documents to avoid duplications, it is necessary to point out that the study is not without limitations. A previous bibliometric study [117], reported some challenges encountered while carrying out bibliometric studies. In the present study, the search was based on article title, abstract, and keyword. This was done to minimize loss of vital documents and to ensure the maximum retrieval of documents associated with the research topic. However, this might introduce false-positive results by delivering closely related topics which are not specific to the research title [115]. Furthermore, given the specificity of the search keywords, articles relevant to the study which were not constructed based on the keywords might be lost. Secondly, the total number of documents used may not reflect 100% accurate information on colistin-resistant *K. pneumoniae* since some articles relating to the field might not all be available on the Scopus database [118]. In addition, to ensure the logical presentation of the retrieved information, authors employed certain benchmarks such as a minimum number of documents, the maximum number of authors per document, and the minimum number of occurrences. Although this was done to identify front liners in every aspect of the study, previous studies have recognized it as a drawback [119]. It is also important to note that the information presented in this study might not necessarily represent the current state of AMR in the various countries and regions of the world, given that the study was not a surveillance of AMR across the regions but was based on published articles from different countries and regions. Hence, it is possible that those countries which failed to publish or whose publications are not indexed in Scopus might be underestimated.

## 4. Materials and Methods

### 4.1. Protocol for Systematic Review

The systematic review and bibliometric analysis were carried out following the PRISMA guidelines [120].

### 4.2. Inclusion Criteria

Data on *K. pneumoniae* resistance to colistin in published scientific papers were accessed from the Scopus Database on 19 July 2020. Initially, the data were obtained from the Web of Science core and Scopus.com to compare the volume and similarity of the documents available on each database. The Scopus database had 1821 published scientific papers relating to the topic, while the Web of Science core yielded 1303 published papers. An outline of the data retrieval process and analysis is presented in Figure 1. Search keywords were colistin-resistant *K. pneumoniae*. The year of publication was set as 1995–2019, and the search was restricted to original research articles with experimental findings relating to colistin resistance in *K. pneumoniae*, excluding reviews, proceedings, communications, abstracts, opinions, and letters. To ensure the retrieval of all articles relating to the subject, the search was broadened and extended to titles, abstracts, and keywords.

### 4.3. Study Selection

The documents were reviewed for duplication, and the abstract of selected papers were evaluated. The studies were independently screened by OZN, and TP based on titles, abstracts, and relevance. Discrepancies were resolved through consultation with the supervisors SPV and CS.

### 4.4. Data Analyses

The 1819 data obtained from the Scopus database were imported into the VOS viewer software and MS Excel for analysis. Data exported to MS Excel were edited, sorted, and categorized based on year of publications, countries, region, and fields, whereas data imported to the VOS viewer were used to create network maps of co-authorships of countries, journal participation, and co-occurrence of authors keywords.

## 5. Conclusions

The present study conducted a bibliometric analysis of published articles relating to colistin-resistant *K. pneumoniae* for a period of 24 years (1995–2019), to help understand the research status and trends relating to the topic. Major findings of the work include:

- A total of 1819 published articles excluding reviews, communications, proceedings, and editorials were retrieved from the Scopus database for the period from 1995 to 2019.
- There is a steep increase in the number of published articles over the last three years (2017–2019), amounting to 50.19% of all the retrieved documents.
- 99 countries had published contributions on colistin resistance in *K. pneumoniae*; however, 26 countries had a minimum of 20 publications and accounted for 82.83% of all the published documents and 91.22% of citations.
- Europe had the highest contribution, followed by America and the West Pacific. Research contributions from the Eastern Mediterranean, Southeast Asia, and Africa were relatively low.
- A total of 455 journals participated in the publications; however, the top 31 active journals accounted for 56.5% of publications and 64.7% of citations.
- International collaborations were highest between America and China and America and Australia. There were also high collaborations between the European nations. International collaborations were low for Africa and Asia.
- Research funders included government agencies, institutions, pharmaceutical industries, and charitable organizations. The United States of America, however, topped the list of funders, whereas no funding agencies were from Africa.

## Figures and Tables

**Table 1:** Reported mechanisms of colistin resistance in *Klebsiella pneumoniae*.

Reference	No of Isolate	Country	Source	Mechanism
[59]	32 CRKP	Lao PDR, Thailand, Nigeria, and France	Clinical	Insertion sequence in *mgrB* gene Missense mutations Mutations in *pmrAB*
[60]	2 CRKP	Italy	Clinical	Single nucleotide polymorphisms Insertions and deletions Specific patterns of gene presence and absence
[61]	4 KP isolate	-	Clinical	Mutations in *crrAB* genes
[62]	25 CRKP	Italy	Clinical	Frameshift mutation and premature termination of *mgrB* gene Non-sense, mutation Insertional inactivation of *mgrB* gene Missense mutations in the *mgrB* gene *phoQ* missense Missense mutation in *pmrA* gene
[63]	13 MDR-KP	Tunisia	Clinical	Modifications (deletions, insertions, and substitutions) in *mgrB*, *pmr*, and *pho* operons
[64]	98 CRKP	-	Clinical	Alterations in the *mgrB* gene
[65]	8CRKP	-	Clinical	Mutation of *eptA* and *arnT* genes Substitutions in *mgrB*, *pmrB*, *phoP* and *phoQ* genes
[66]	23 CRKP	Saudi Arabia	Clinical	Mutations in *mgrB* or *PhoP* genes Presence of ISKpn14 is the major IS while ISKpn28
[67]	17 CRKP	Oman	Clinical	Insertion-sequences in *mgrB* gene
[37]	5 Col-R KP	Laos & Thailand	Clinical	Presence of plasmids IncP1, IncFII, and IncI1 with *mcr-3* gene IncFII type plasmid with *mcr-8* gene
[68]	15 KP	Israeli	Clinical	Missense mutation in the *mgrB* gene Inactivation of mgrB by an IS5-like insertion sequence
[69]	NDM-5 CRKP	Lebanese	Clinical	Mutations in the amino acid sequences of proteins (PmrB, PhoQ, and MgrB)
[70]	65 KP isolates	India	Clinical	Mutations in *mgrB* Premature stop codon at 21st amino acid Presence of insertion sequences (IS903, IS Kpn 14 and ISK pn 26) Elongation of *mgrB* Mutations were also observed among *phoP* and *phoQ* genes.
[53]	3 Col-R KP	Bangladesh	Clinical	Presence of IncFIB(pQil) plasmid with *mcr-8.1*
[71]	45 Col-R KP	Serbia	Clinical	Mutation MgrB IS5-mediated inactivation
[72]	1 Col-R KP	-	Chicken	Presence of *mcr-8.2* and *mcr8*-bearing plasmid
[73]	2 Col-R KP	Malaysia	Neonate	Interruption of *mgrB* gene by insertion sequences
[30]	49 Col-R KP	Taiwan	-	*mgrB* gene alterations Variations in *crrB*, *pmrB*, *phoQ*, *pmrA*, and *phoP* genes. Presence of insertion sequence, ISKpn26, ISEcp1, IS10R, IS903B or ISKpn14 in *mgrB* or crrCAB region
[74]	1 isolate KP65	China	Clinical	Insertion of IS5 at position 117 of *mgrB* gene
[75]	71 Col-R KP	Russia	Clinical	Inactivation of *mgrB* by insertion sequences IS1A, IS1R, ISKpn14, and ISKpn26 Novel miniature inverted-repeat transposable element (MITE) Kpn1
[76]	65 MDR KP	Poland	Clinical	Presence of *mcr-1* gene
[51]	K9 isolate	Lebanon	Clinical	Presence of *mcr-8* gene
[77]	4 KPC-2 KP	India	Clinical	Overexpression of *acrB*, *tolC*, *ramA*, and *soxS* genes of the AcrAB-TolC pump system
[29]	213 Col-R KP	Greece	Clinical	Inactivation of the *mgrB* gene including insertion sequences. nonsense mutations missense mutations
[54]	4 COL-R KP	China	Clinical	Presence of *mcr-8.2*
[78]	27 CRCR-KP	Italy	Clinical	Variations in *mgrB*, *phoQ*, and *pmrB*
[41]	3 KP isolates	Czech Republic	Turkey meat	Presence of the *mcr-1* gene
[79]	4 KP isolates	Portugal	Pig farms	Presence of the *mcr-1* gene
[80]	14 KP isolates	Croatia	Clinical	DNA mutations causing mutations in the MgrB protein
[14]	2 KP isolates	Egypt	Clinical	Presence of the *mcr-1* gene *mgrB* mutations
[42]	-	China	Retail fruits	Presence of *mcr-1* gene
[81]	2 KP isolates	China	Fishery,	Presence of *mcr-1* gene
[82]	15 KP isolates	Brazil	Clinical	Mutations in *pmrB* gene
[83]	KP ST101	Brazil	Clinical	Presence of *mcr-1* gene
[38]	2 KP isolates	Iran	Clinical	Presence of *mcr-1* gene
[11]	46 KP isolates	Malaysia	Swine	Presence of *mcr-1* gene
[84]	-	Vietnam	Clinical	Presence of *mcr-1* gene Modifications of proteins involved in lipopolysaccharide (PmrA, PmrB, PmrC, PmrI, and PmrJ)
[43]	10 KP isolates	Indian	Food samples	Alterations in *mgrB*
[85]	-	China	Retailed Raw Meat	Presence of IncX4 plasmid carrying *mcr-1* gene
[86]	20 KP isolates	Italy	Clinical	Mutations and insertion elements in *mgrB*
[87]	20 COL-R KP	-	-	Increased expression of the *pmrA*, *pmrB*, *pmrD*, *pmrK*, *phoP*, and *phoQ* genes
[88]	1 KP isolate	São Tomé and Príncipe	Clinical	Presence of plasmid-borne *mcr-1* gene
[89]	3 KP isolates	China	Clinical	Inactivation of PmrAB or MgrB Mutation of CrrB (E189K)
[90]	1 KP isolate	China	Clinical	ISKpn26-like insertion in the disrupted *mgrB* gene
[91]	-	Japan	Clinical	Mutation in the *mgrB* gene
[92]	-	Lebanon	Clinical	Amino acid substitutions into protein sequences of *pmrA/B*, *phoP/Q*, and *mgrB*
[16]	-	USA	Clinical	Insertion sequence disruption was limited to *mgrB*
[93]	11 COL-R KP	South Korea	Clinical	Amino acid substitutions were identified in PmrAB, PmrD, PhoPQ, and MgrB
[31]	15 COL-R KP	Greece	Clinical	Insertion of ISKpn26-like element at nucleotide position 75 of mgrB,
[94]	-	Algeria	Clinical	Amino acid substitutions in PmrA/B and a truncated *mgrB* gene
[95]	5 KP isolates	China	Clinical	Inactivation of the *mgrB* gene
[55]	5 KP isolates	China	Chicken	Plasmid co-harbouring both tmexCD1-toprJ1 pump and *mcr*
[96]	-	China	Clinical	Missense mutation in *crrB* gene Presence of multidrug efflux pump KexD
[97]	-	-	Clinical	Missense mutations of *crrB* gene Increased expression of H239_3064 (RND-type efflux pump)
[98]	-	USA	Clinical	Increased expression of efflux pump AcrAB-TolC Reduced expression of ompK35 and ompK37
[99]	11 KP isolates	India	Clinical	SMR-type efflux pump (KpnEF)

*MCR*: Mobilized colistin resistance, NDM: New Delhi metallo-beta-lactamase, MDR: Multi-drug Resistance, IS: Insertion sequence, ST: Sequence type, MIC: Minimum inhibitory concentration, CTX: cefotaxime-beta lactamases, OXA: Oxacillinase, RND: Resistance-nodulation-division, CRKP: Carbapenem-resistant *Klebsiella pneumoniae*; KP: *Klebsiella pneumoniae*, Col-R: Colistin resistant, OMP-Outer membrane protein.

**Table 2:** Reported prevalence of colistin resistance in *Klebsiella pneumoniae* isolates.

Reference	Prevalence (%)	Source/Site	Country	Study Year
[100]	10.9, 25.6, and 21.9	Clinical and Surveillance rectal swab	Italy	2012–2014
[101]	0.4	Clinical isolates	Spain	2012–2015
[18]	76.1	Clinical CRKP isolates	Thailand	2014–2017
[102]	1.5	Clinical isolates	USA, Europe, Turkey, Israel, Latin America Asia-Pacific	2006–2009
[103]	0.6	Clinical isolates	China	2011–2014
	23.4	Avian isolates	China	2013
[104]	33.3	Food animals	China	2016
[105]	0.96	Poultry isolates	Iran	2017–2018
[106]	-	Clinical isolates	China	2015–2016

## Data Availability

Not applicable.

## Supplementary Materials

The following are available online at https://www.mdpi.com/article/10.3390/diseases9020044/s1, Figure S1: Overlay of countries, showing countries with the most currently published articles relating to colistin resistance in K. pneumoniae.

## Abbreviations

MCR: Mobilized colistin resistance; NDM: New Delhi metallo-beta-lactamase; MDR: Multi-drug Resistance; IS: Insertion sequence; ST: Sequence type; MIC: Minimum inhibitory concentration; CTX: cefotaxime-beta lactamases; OXA: Oxacillinase, RND: Resistance-nodulation-division; CRKP: Carbapenem-resistant *K. pneumoniae*; KP: *K. pneumoniae*; Col-R: Colistin-resistant; EUR: European Region; AMR: America Region; WPR: West Pacific Region; EMR: Eastern Mediterranean Region; SEAR: South East Asia Region; AFR: Africa Region.
