# Supplementary material for: A Bibliometric Meta-Analysis of Colistin Resistance in Klebsiella pneumoniae
Source: Diseases. 2021 Jun 20;9(2):44. doi: 10.3390/diseases9020044 (PMC8293170; doi:10.3390/diseases9020044)
Supplement: Supplementary file 1 [file diseases-09-00044-s001.zip › diseases-1232194-supplementary.pdf]

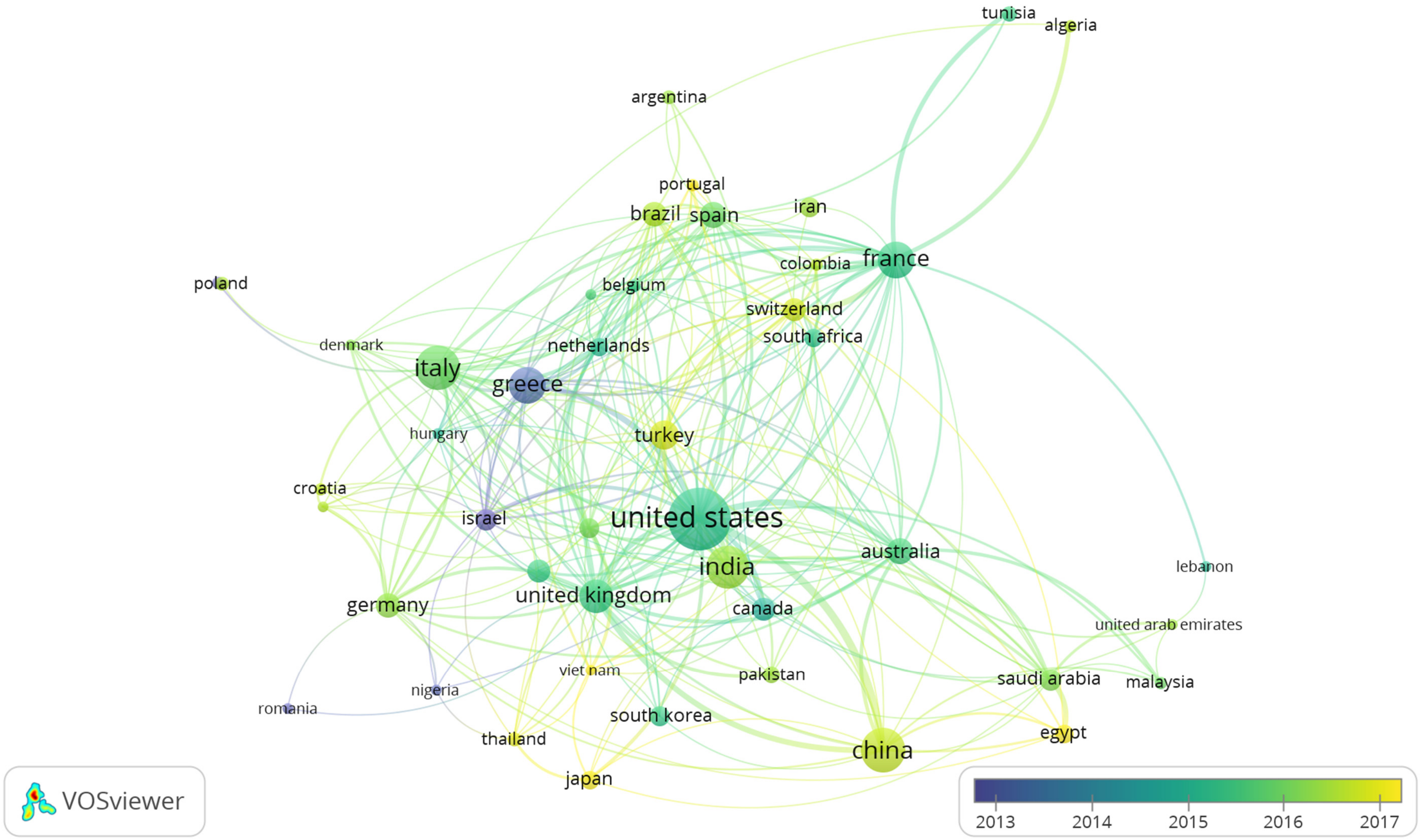

Figure S1. Overlay of countries, showing countries with the most currently published articles relating to colistin resistance in *K. pneumoniae*.
